# *In Vitro* Effect of *Porphyromonas gingivalis* Methionine Gamma Lyase on Biofilm Composition and Oral Inflammatory Response

**DOI:** 10.1371/journal.pone.0169157

**Published:** 2016-12-29

**Authors:** Abish S. Stephen, Emma Millhouse, Leighann Sherry, Joseph Aduse-Opoku, Shauna Culshaw, Gordon Ramage, David J. Bradshaw, Gary R. Burnett, Robert P. Allaker

**Affiliations:** 1 Research Centre for Clinical & Diagnostic Oral Sciences, Blizard Institute, Queen Mary University of London, London, United Kingdom; 2 Infection and Immunity Research Group, Dental School, University of Glasgow, Glasgow, United Kingdom; 3 GlaxoSmithKline Consumer Healthcare, Weybridge, United Kingdom; Oregon Health & Science University, UNITED STATES

## Abstract

Methanethiol (methyl mercaptan) is an important contributor to oral malodour and periodontal tissue destruction. *Porphyromonas gingivalis*, *Prevotella intermedia* and *Fusobacterium nucleatum* are key oral microbial species that produce methanethiol via methionine gamma lyase (*mgl*) activity. The aim of this study was to compare an *mgl* knockout strain of *P*. *gingivalis* with its wild type using a 10-species biofilm co-culture model with oral keratinocytes and its effect on biofilm composition and inflammatory cytokine production. A *P*. *gingivalis mgl* knockout strain was constructed using insertion mutagenesis from wild type W50 with gas chromatographic head space analysis confirming lack of methanethiol production. 10-species biofilms consisting of *Streptococcus mitis*, *Streptococcus oralis*, *Streptococcus intermedius*, *Fusobacterium nucleatum* ssp *polymorphum*, *Fusobacterium nucleatum* ssp *vincentii*, *Veillonella dispar*, *Actinomyces naeslundii*, *Prevotella intermedia* and *Aggregatibacter actinomycetemcomitans* with either the wild type or mutant *P*. *gingivalis* were grown on Thermanox cover slips and used to stimulate oral keratinocytes (OKF6-TERT2), under anaerobic conditions for 4 and 24 hours. Biofilms were analysed by quantitative PCR with SYBR Green for changes in microbial ecology. Keratinocyte culture supernatants were analysed using a multiplex bead immunoassay for cytokines. Significant population differences were observed between mutant and wild type biofilms; *V*. *dispar* proportions increased (p<0.001), whilst *A*. *naeslundii* (p<0.01) and *Streptococcus* spp. (p<0.05) decreased in mutant biofilms. Keratinocytes produced less IL-8, IL-6 and IL-1α when stimulated with the mutant biofilms compared to wild type. Lack of *mgl* in *P*. *gingivalis* has been shown to affect microbial ecology *in vitro*, giving rise to a markedly different biofilm composition, with a more pro-inflammatory cytokine response from the keratinocytes observed. A possible role for methanethiol in biofilm formation and cytokine response with subsequent effects on oral malodor and periodontitis is suggested.

## Introduction

Sulfur metabolism is an indispensable part of the biological processes in the biosphere [1, 2]. Among the enzymes involved in sulfur metabolic pathways, a group of carbon-sulfur lyases namely, methionine γ lyases (EC. 4.4.1.11) are thought to be unique to a few genera within the tree of life but not found in humans [3]. This group of pyridoxal 5’-phosphate dependent enzymes are thought to catalyze a number of reactions including α, γ-elimination of L-methionine and α, β-elimination of L-cysteine, yielding α-keto acids, ammonia and thiols [4]. In bacteria, methionine γ lyase is thought to be involved in anaerobic energy metabolism and the ability of this enzyme to generate volatile sulfur compounds (VSCs) as byproducts has been applied in forming flavors in the fermented food industries [5].

The anaerobic gram-negative bacterium *Porphyromonas gingivalis* has been well studied in relation to plaque-induced inflammatory diseases of the mouth, and is one of the few oral organisms known to possess methionine γ lyase (*mgl*) [6]. Classically part of a trio of microbial species (‘red complex’) including *Tanerella forsythia* and *Treponema denticola* that were strongly linked to the etiopathogenesis of periodontitis, *P*. *gingivalis* has now been proposed as a ‘keystone’ pathogen in the new paradigm of periodontitis[7]. This model recognizes that some bacterial species though in low abundance are able to drive the ecology of the biofilm community in response to environmental cues or adaptations by influencing the diversity and evenness of such communities. Studies have elaborated the mechanisms by which *P*. *gingivalis* could bring about changes in the community structure by manipulating host response. These include interfering with neutrophil recruitment by deactivating IL-8 production, down regulation of E-selectin production by gingival epithelia, impeding the complement cascade by effecting C5aR/TLR2 crosstalk and gingipain induced degradation of important complement proteins such as C3 and C5[8–11]. Whilst these mechanisms could elicit a sustained, if ineffective immune response from the host, the adaptation of *P*. *gingivalis* to the subgingival habitat is thought to be realized in the episodic nature of these mechanisms.

*P*. *gingivalis* is known to produce VSCs in serum and also from free sulfur containing amino acids such as cysteine and methionine; however it is reported to be more efficient at producing VSCs from serum substrate than cysteine and methionine [12, 13]. These VSCs such as hydrogen sulfide, methanethiol and dimethyl disulfide are thought to be useful in lowering the redox potential of the subgingival microenvironment and aid in the invasiveness of the organism and indeed the biofilm, by increasing the permeability of the mucosal cell membrane [14]. Given that one of the described mechanisms of IL-8 deactivation by serB serine phosphatase requires cell invasion for it to occur [10], one would expect VSC production to be an important adaptive mechanism for *P*. *gingivalis* and indeed for other potential keystone pathogens.

Of the genetic complement that can produce VSCs in *P*. *gingivalis*, a previous study had characterized the methionine Ɣ lyase (*mgl*) that produces methanethiol by catabolic degradation of methionine, and this enzyme has been reported to confer resistance to the antimicrobial agent 3-chloro-DL-alanine by exhibiting deaminating activity towards the antimicrobial agent as with methionine, in both *P*. *gingivalis* and *F*. *nucleatum* [6, 15]. A virulence study in a murine model demonstrated that *P*. *gingivalis* W83 wild type was markedly more invasive than the *mgl*-deficient mutant, consistent with the hypothesis that VSC production could aid invasiveness [6]. However, it is well known that planktonic forms of bacteria often exhibit a different phenotype to biofilms and these studies did not involve biofilms and it is possible biofilm dwelling *P*. *gingivalis* downregulates VSC producing genes to exhibit a less invasive phenotype. The present study aimed to replicate the knockout of the *mgl* in the strain W50 (as opposed to W83). Then, the mutant and wild type *P*. *gingivalis* were grown in a 10-species oral biofilm model and used to stimulate transformed oral keratinocytes *in vitro*. Biofilm composition was determined to study the effect of the *mgl* gene knockout in influencing the ecology of the biofilm, and if the possible altered ecology in any way affects the cytokine response of the oral keratinocytes.

## Materials and Methods

### Bacterial strains and culture conditions

*Porphyromonas gingivalis* W50 was maintained on Fastidious Anaerobe agar (+5% v/v defibrinated horse blood; FAA) in an anaerobic atmosphere (80% N_2_, 10% H_2_ and 10% CO_2_) at 37°C. Liquid culture of *P*. *gingivalis* was carried out in Brain Heart Infusion (3.7% w/v; BHI) broth supplemented with 0.0005% w/v Hemin.

Agar used in culture of organisms for constructing 10-species biofilms were FAA and Columbia Blood agar (+5% v/v defibrinated horse blood; CBA). Broths used were Schaedler’s Anaerobic broth (SCH), BHI and Tryptic Soy broth (+0.8% w/v glucose and 0.6% w/v yeast extract; TSB). In maintaining and building up the 10-species biofilms, the medium Artificial Saliva (AS) was exclusively used [16].

Bacterial strains were: *Porphyromonas gingivalis* W50 (FAA; SCH), *Streptococcus mitis* NCTC 12261 (CBA; TSB), *Streptococcus intermedius* DSM 20573 (CBA; TSB), *Streptococcus oralis* NCTC 11427 (CBA; TSB), *Aggregatibacter actinomycetemcomitans* ATCC 43718 (CBA; TSB), *Veillonella dispar* NCTC 11831 (FAA; BHI), *Actinomyces naeslundii* DSM 17233 (FAA; BHI), *Fusobacterium nucleatum* ssp *polymorphum* ATCC 10953 (FAA; SCH), *Fusobacterium nucleatum* ssp *vincentii* DSM 19507 (FAA; SCH), *Prevotella intermedia* DSM 20706 (FAA; BHI).

*Streptococcus* spp. and *A*. *actinomycetemcomitans* were maintained and cultured in an aerobic atmosphere containing 5% CO_2_, whilst all other strains were maintained in an anaerobic atmosphere (80% N_2_, 10% H_2_ and 10% CO_2_) at 37°C.

### Generation of *mgl*-deficient *P*. *gingivalis*

The open reading frame (ORF) coding for *m*ethionine *g*amma *l*yase (*mgl*) in *P*. *gingivalis* W50 was identified as per the previous study [6]. The ORF was identified and amplified by using the oligonucleotide primers listed in Table 1 (irrelevant sequence in italics and restriction enzyme recognition sequence in bold).

**Table 1:** Primer pairs complementary to the bacterial 16S gene used in quantifying bacterial biofilm populations in this study and the primer pairs used for mutagenesis.

Bacteria	Primer sequences	Reference
*Streptococcus* spp.	5’-GATACATAGCCGACCTGAG-3’	[20]
	5’-CCATTGCCGAAGATTCC-3’	
*A*. *naeslundii*	5’-GGCTGCGATACCGTGAGG-3’	[20]
	5’-TCTGCGATTACTAGCGACTCC-3’	
*Veillonella* spp.	5’-CCGTGATGGGATGGAAACTGC-3’	[21]
	5’-CCTTCGCCACTGGTGTTCTTC-3’	
*A*. *actinomycetemcomitans*	5’-GAACCTTACCTACTCTTGACATCCGAA-3’	[22]
	5’-TGCAGCACCTGTCTCAAAGC-3’	
*P*. *gingivalis*	5’-GGAAGAGAAGACCGTAGCACAAGGA-3’	[23]
	5’-GAGTAGGCGAAACGTCCATCAGGTC-3’	
*Prevotella* sp	5’-CGGTCTGTTAAGCCTGTTGTG-3’	[24]
	5’-CACCATGAATTCCGCATACG-3’	
*Fusobacterium* spp	5’-AAGCGCGTCTAGGTGGTTATGT-3’	[25]
	5’-TGTAGTTCCGCTTACCTCTCCAG-3’	
Universal	5’-GTGSTGCAYGGYTGTCGTCA-3’	[26]
	5’-ACGTCRTCCMCACCTTCCTC-3’	
PG0343F1	5’-CATAGACGATCCTCGGTCG-3’	
PG0343R1	5’-*atatat***gagctc**ATATTGGGGTTGGCCGGAG-3’ (SstI)	
PG0343F2	5’-*atatat***tctaga**TCACGGGGGCCAATATGAG-3’ (XbaI)	
PG0343R2	5’-TGTCCTCCACGTTCTCCAG -3’	

The amplicons were purified (Qiagen) after their sizes were confirmed by agarose gel electrophoresis and digested with the respective *Sstl* and *XbaI* enzymes (NEB). The digested amplicons were then purified and ligated to a pre-digested *ermF-ermAM* cassette from the plasmid pVA2198 [17]. The ligation mixture was then purified and the product reamplified (ReddyMix Extensor PCR MasterMix 1), with the resulting ~3kb fragment used to electroporate exponential growth phase *P*. *gingivalis* W50 cells (Bio-Rad Gene Pulser) to promote mutagenesis by allele exchange. Following overnight recovery under anaerobic conditions, the cell suspension aliquots were plated on to Blood Agar (containing 5μg/mL clindamycin-HCl) and incubation was allowed to continue, anaerobically. The resultant colonies were screened by PCR and products visualized via agarose gel electrophoresis to confirm incorporation of the *ermF-ermAM* cassette. The mutant strain in this paper will be referred to as PG343.

#### Enzyme assays

Spectrophotometric assays of the arginine and lysine gingipain activity of the wild type and mutant strains were carried out as described elsewhere [18]. Briefly, *P*. *gingivalis* W50 and PG343 were grown in BHI-Hemin broth up to an OD_600_ of 0.8 before obtaining the culture supernatant after centrifugation at ~7000 *g* for 2 mins. A 10μl sample of culture or culture supernatant were used to catalyze breakdown of substrates for arginine and lysine proteases, benzoyl-Arg-p-nitroanilide (Sigma) and N-α-acetyllysine-p-nitroanilide (Bachem), respectively in cuvettes with a 1cm light path at 30°C. Absorbance was monitored at 405nm.

### Gas chromatographic headspace analysis

*P*. *gingivalis* suspensions (OD_600_ = 0.1) were prepared by pelleting 48 hour anaerobic broth cultures at 3000 *g* for 5 mins and washing the cells in Phosphate Buffered Saline (Sigma; pH 7.2±0.2) twice, before resuspending in PBS. 0.3mL of the cell suspension was added to 0.5mL of 0.5% w/v L-methionine solution in a 10mL headspace vial, sealed and incubated at 37°C for 1 hour with shaking (140 rpm) before adding 1ml of absolute ethanol (Sigma) to arrest bacterial metabolism of substrate.

The Gas Chromatograph (Agilent G6890N, Agilent Technologies, Edinburgh, UK) was calibrated by CH_3_SH and H_2_S gas standards generated from permeation tubes by a gas standards generator (Kin-tek 491M).

The headspace of each vial was sampled manually using a gas tight syringe (NORM-JECT^®^, Henke-Sass Wolf) after incubating at 80°C for 2 minutes. 250μl of the headspace sample was introduced on to a Chromosil 330 packed column (8’ x 1/8” OD Teflon^®^ (FEP) tubing with central 6’ packed) through a sulfinert-treated sample loop connected to a sampling valve with helium carrier gas flowing through the column at a constant flow rate of 45ml/min, via an inlet at 120°C. The packed column was maintained throughout the runs isothermally at 60°C. The Flame Photometric Detector was maintained at 175°C with H_2_ and air flow at 50 ml/min and 75 ml/min respectively, with N_2_ makeup gas at flow rate of 15ml/min.

### Preparation of ten-species biofilms

Ten-species biofilms were built up on Thermanox coverslips as detailed elsewhere [16, 19] in the following order: an initial 3-species biofilm consisting of *S*. *mitis*, *S*. *intermedius* and *S*. *oralis* were inoculated with standardized AS suspensions of *V*. *dispar* (BHI), *A*. *naeslundii* (BHI), *F*. *nucleatum* ssp *polymorphum* (SCH) and *F*. *nucleatum* ssp *vincentii* (SCH) anaerobically overnight at 37°C to obtain an intermediate 7-species biofilm. This biofilm was further inoculated with AS suspensions of *P*. *gingivalis* (SCH), *P*. *intermedia* (BHI) and *A*. *actinomycetemcomitans* (TSB) and the resulting 10-species biofilm allowed to mature for 4 days with renewal of the AS medium every 24 hrs. After this maturation phase the biofilms were stored at -80°C till use.

#### Stimulation of keratinocyte cell line

The immortalized oral keratinocyte cell line OKF6-TERT2 was grown as a monolayer in 24-well cell culture plates and challenged with the 10-species biofilms grown with the wild type or mutant *P*. *gingivalis* for 4h and 24h as described previously [16]. Six stimulations were performed on three occasions using independent batches of biofilms, with the supernatant samples pooled to obtain triplicate samples per batch.

#### qPCR analysis of biofilms

Triplicate biofilms grown thrice independently, were used for biofilm composition study. Biofilms on the Thermanox coverslips were revived in AS overnight and the biofilms washed with PBS (pH 7.2±0.2) thrice before disrupting the biofilms by sonication for 10 minutes in PBS. DNA extraction from the recovered sonicate was performed as described previously [16] using the MasterPure GramPositive DNA purification kit (Epicentre Biotechnologies, Madison, USA).

qPCR was performed by using a SYBR Green I fluorophore (Roche) in the LightCycler 480 (Roche). Assays were performed in 20μL total volume per well in 96 well plates. Recommended thermal cycling conditions were used: initial denaturation at 95°C for 5 mins followed by 40 cycles of 10s at 95°C, 5s at 55°C, 30s at 72°C and 1s at 76°C. Standard curves were obtained by running decimal dilutions of bacterial DNA extracted from pure cultures with known CFU mL^-1^. Genomic DNA extracted from *P*. *gingivalis* cultures were used to generate standard curves for the universal primer. The data calculated as CFU from the qPCR analysis for general bacterial load and the specific bacteria were then converted into proportions for statistical analysis. Primer pairs used in detection of the species used in the biofilm model are listed in supporting information Table 1.

#### Multiplex immunoassay for cytokine analysis

Cell culture supernatants were collected at the end of biofilm stimulation and cytokines measured in 100μL of each sample by a multiplexed bead immunoassay (FlowCytomix, eBioscience) whereby the samples were prepared according to the kit manufacturer’s instructions. Standard curves were setup with the reagents supplied by the manufacturer with the samples and standards analysed with a flow cytometer (BD FACSCanto II). The raw data was then processed using the kit manufacturer’s software (FlowCytomix Pro v3.0) to determine the concentration of twenty cytokines: E-selectin, G-CSF, ICAM-1, TGF-β, IFN-α, IFN-γ, IL-1α, IL-1β, IL-4, IL-6, IL-8, IL-10, IL-12p70, IL-13, IL-17a, CXCL10, MCP-1, MIP-1α, MIP-1β and TNF-α.

#### mRNA analysis by qPCR array

After collection of the cell culture supernatant, the monolayer of cells were lysed and RNA extracted using the RNeasy kit (Qiagen), using the provided spin columns, and performing on-column DNase digestion. Equimolar quantities of RNA (280 ng) from all samples were converted to cDNA with the RT^2^ First-Strand kit (Qiagen) and then rt-PCR was conducted in a custom 384-well array format in the LightCycler 480 (Roche) with RT^2^ SYBR Green qPCR MasterMix (Qiagen). Cytokines measured were TNF-α, IL-1α, IL-6, IL-8, IL-18, IL-13, MIF, CCL20 and CXCL10. Reference genes used to normalize measured Ct values were Actin-β, Glyceraldehyde-3-phosphate dehydrogenase (GAPDH) and hypoxanthine phosphoribosyltransferase 1 (HPRT1). Relative gene expression was calculated and expressed as ΔCt relative to the most stable reference gene, while genomic DNA contamination, RT-PCR efficiency and positive PCR controls run for each sample in the array.

### Statistical analyses

All statistical analyses were performed using the software GraphPad Prism 6.0 (GraphPad Software Inc, La Jolle, USA) for Microsoft^®^ Windows. Non-parametric statistical tests such as Kruskal-Wallis and Mann Whitney U tests were used in analyzing for differences between experimental groups.

## Results and Discussion

Methanethiol formation by *Porphyromonas gingivalis* strain W83 was studied previously and disabling the enzyme responsible for methanethiol production, namely methionine Ɣ lyase (*mgl*), was found to reduce the pathogenicity of the bacterium in a murine model [6]. The present study investigated the possibility that *mgl* of *P*. *gingivalis* may have roles to play in influencing the biofilm composition and hence, cytokine response of the oral epithelium. As such, we first mutated *mgl* in the *P*. *gingivalis* strain W50 to replicate the previous work in a novel strain, and then used it in a 10-species biofilm co-culture model (‘Glasgow Model’).

### VSC production and gingipain activities of PG343

Gas chromatographic headspace analysis of the mutant and wild type strains in planktonic suspensions revealed that PG343 produced little or no methanethiol compared to the W50 given equal amounts of L-methionine, suggesting knockout. However, it is possible that *P*. *gingivalis* W50 has other as yet discovered gene(s) still capable of producing methanethiol, as the previous study on strain W83 observed complete absence of methanethiol in the headspace of bacterial cells incubated with L-methionine after mutagenesis but not in the culture supernatants [9]. Interestingly, we observed that the amount of H_2_S generated in the headspace by PG343 from L-cysteine was approximately 40% of the concentrations in the headspace of W50. It is possible that the *P*. *gingivalis mgl* is also able to catalyze α, β-elimination of L-cysteine and this function is also reduced in PG343.

Colorimetric assays showed similar activities for both arg-gingipain (Rgp) and lys-gingipain (Kgp) in PG343 and W50 broth cultures and supernatants. As reported in the literature, the culture supernatants of W50 and PG343 showed marked reduction in measured enzyme activity for both the gingipains, but this was particularly evident for Kgp [27], and this possibly suggests that virulence dependent on the gingipains has been preserved in this strain. No differences could be observed in the colony morphology between W50 and PG343, with both strains acquiring black pigmentation after similar anaerobic incubation times on BA.

### Effect of *P*. *gingivalis mgl* on multi-species biofilm composition

**Fig 1:**
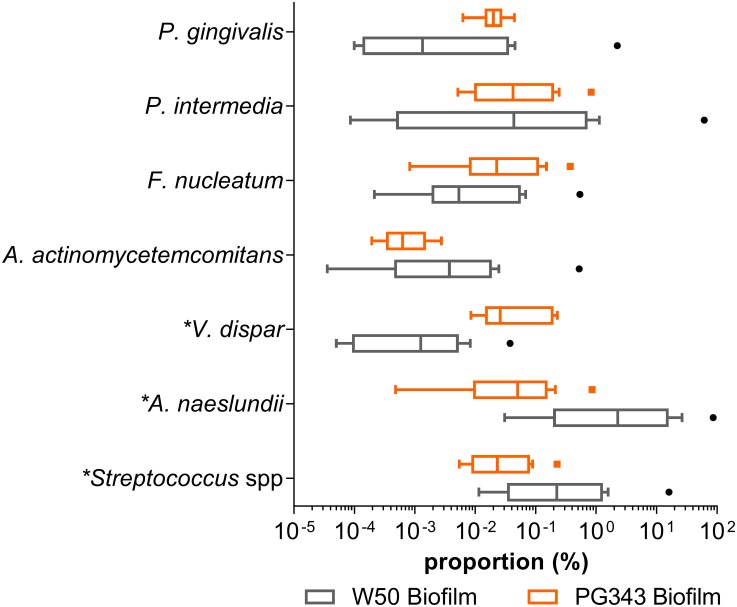
The proportions of the different species in the PG343 and W50 biofilms. n = 9 for both PG343 and W50 biofilms. Proportions are relative to total CFEs as measured by universal primers in each biofilm. Boxes extend from 25th to 75th percentile, mid line denotes median. Whiskers and outliers plotted by the Tukey method. Asterisk before species name indicates statistical significance.

**Fig 2:**
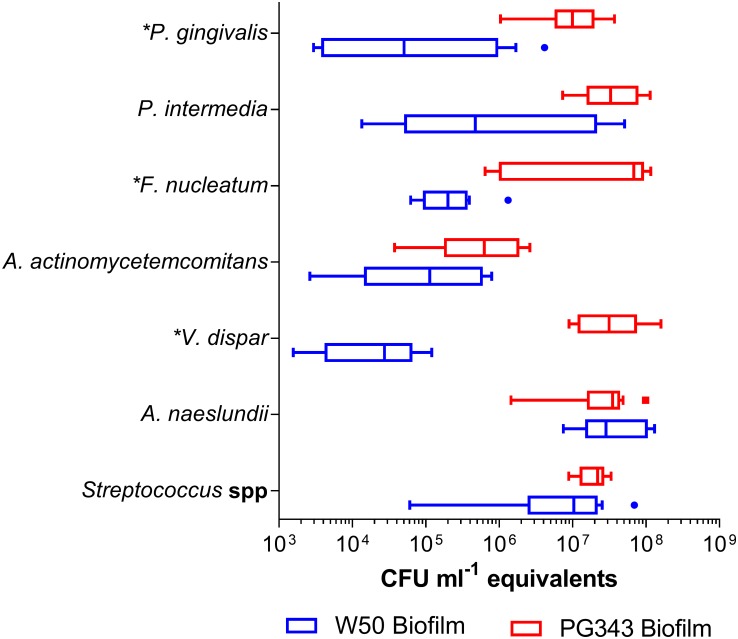
The composition of the W50 and PG343 biofilms. CFU ml^-1^ normalized to the amounts of DNA extracted from each biofilm (n = 9 for both PG343 and W50 biofilms). p<0.01 for *F*. *nucleatum*, *V*. *dispar* and *P*. *gingivalis*. Boxes extend from 25th to 75th percentile, mid line being median. Whiskers and outliers plotted by the Tukey method. Asterisk before species name indicates statistical significance.

Growth of biofilms with PG343 and W50 resulted in markedly different biofilm compositions (Fig 1). *Streptococcus* spp (p = 0.02) and *A*. *naeslundii* (p = 0.004) proportions decreased significantly in the mutant biofilms compared to wild type, whereas *V*. *dispar* proportions increased significantly (p = 0.0008). No statistical differences in the proportions of other species were detected. However, when the amounts of DNA extracted from each sample is taken into account (an indication of biomass) without normalizing to the universal primer set, *F*. *nucleatum*, *V*. *dispar* and *P*. *gingivalis* were found to be significantly increased in the PG343 biofilm compared to W50 (Fig 2). Although not statistically significant, elevated colony forming unit equivalents (CFEs) of *P*. *intermedia* were also observed in the PG343 biofilms compared to W50. Measurement of the total CFEs in the biofilms by using *P*. *gingivalis* cultures as standards indicated that the PG343 biofilms had, on average an order of magnitude more CFEs than the W50 biofilms. The proportions of the individual species in the PG343 biofilms were congruent, resulting in a more even community, whereas the proportions of *Streptococcus* spp and *A*. *naeslundii* were significantly higher relative to the other species in the W50 biofilms.

It is well known that *P*. *gingivalis* is one of the most efficient degrader of free L-methionine and in the form of serum peptides [12]. It is possible that methionine degradation by *P*. *gingivalis* prevents other species, in particular, *F*. *nucleatum* from proliferating by using it as an energy source [28, 29]. It may well be that in the PG343 biofilms, more availability of L-methionine encourages growth of *F*. *nucleatum*, which can further support the proliferation of all the other species as *F*. *nucleatum* is better known as a bridging-species in the oral biofilm [30, 31]. This could explain the increased overall CFEs, and in particular *P*. *gingivalis*, *P*. *intermedia*, *A*. *actinomycetemcomitans* and *V*. *dispar* as measured in the PG343 biofilms. This hypothesis would suggest that *mgl* of *P*. *gingivalis* could have a role to play in the overall ecology and community structure of the oral biofilm, as a keystone species [7]. Indeed, if the increased proportions of *Streptococcus* spp and *A*. *naeslundii* in the W50 biofilms may be thought of as beneficial, owing to their prior associations to healthy plaque [32–34], the presence of *P*. *gingivalis* in low levels with an active *mgl* in the oral biofilm may help regulate the community structure towards a more health associated ecology.

The species that displayed the most substantial change in proportions and CFEs (increase of ~10^3^ CFE) in the PG343 biofilms was *V*. *dispar*. This species lacks *mgl* and it is possible that *V*. *dispar* benefits the most from the *F*. *nucleatum* vs *P*. *gingivalis* dichotomy in the ability to degrade methionine within the present *in vitro* model. The proportions of *P*. *gingivalis* measured in both the PG343 and W50 biofilms did not show a difference (Fig 1), in contrast, an increase in the *P*. *gingivalis* CFEs was measured with the PG343 biofilms (Fig 2), further supporting the explanation that this is possibly an effect of the increased numbers of *F*. *nucleatum* able to support more biomass [35].

### Effect of the biofilms on cytokines released by oral keratinocytes

**Fig 3:**
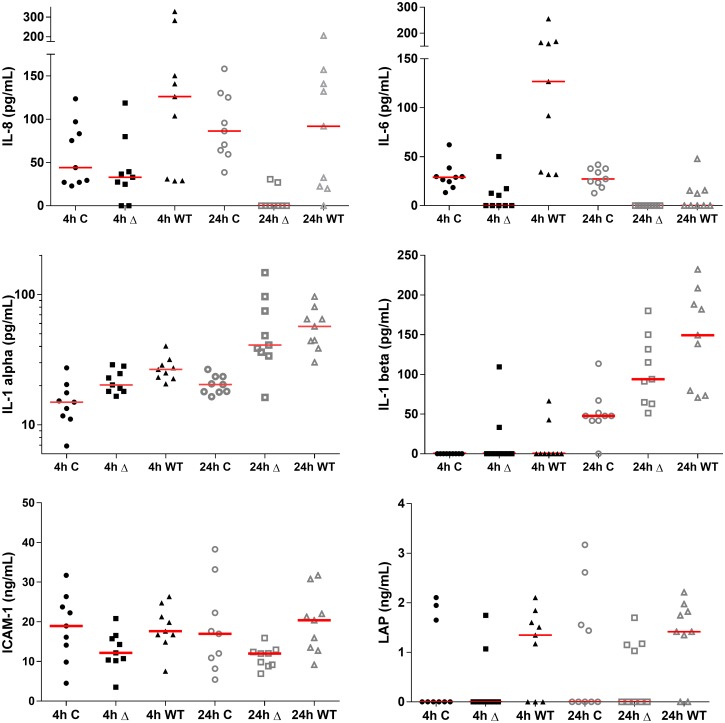
Cytokines that showed significant differences between the PG343 and W50 biofilm stimulations at 4h and 24h. PG343 = Δ; W50 = WT; C = Controls. Median is denoted by the red line.

Analysis of the cell culture supernatants by flow cytometry revealed that the supernatants in the mutant biofilm stimulations contained significantly lower IL-8 in both the 4h (p = 0.03) and 24h (p = 0.005) conditions, whilst the same was observed for IL-6 in just the 4h stimulations (p = 0.0002; Fig 3). Statistical differences with a similar pattern were found between the mutant and wild type biofilm stimulations in: IL-1α at 4h (p = 0.05); ICAM-1 at 4h (p = 0.02) and 24h (p = 0.004); Latency Associated Peptide (TGF-β) at 24h (p = 0.03). No significant differences were observed with the concentrations of interferon-ɣ (IFN-γ), IL-13 or E-selectin between the PG343 and W50 biofilm stimulations (S1 Fig). Except IL-8 and IL-6, amounts of other cytokines tended to increase at 24h vs 4h.

**Fig 4:**
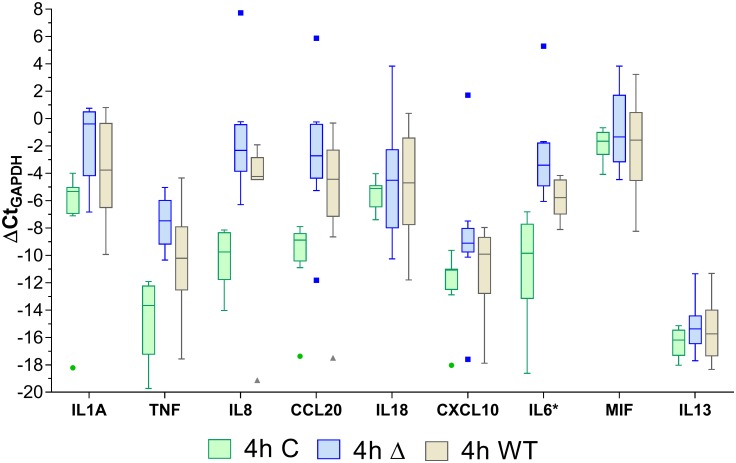
The relative mRNA expression of the stimulated cells and unstimulated controls at the 4h time point. Whiskers and outliers determined by the Tukey method. C = Control; Δ = PG343 biofilms; WT = W50 biofilms. Asterisk after cytokine name in the x-axis denotes statistical significance at p<0.05 between Δ and WT.

**Fig 5:**
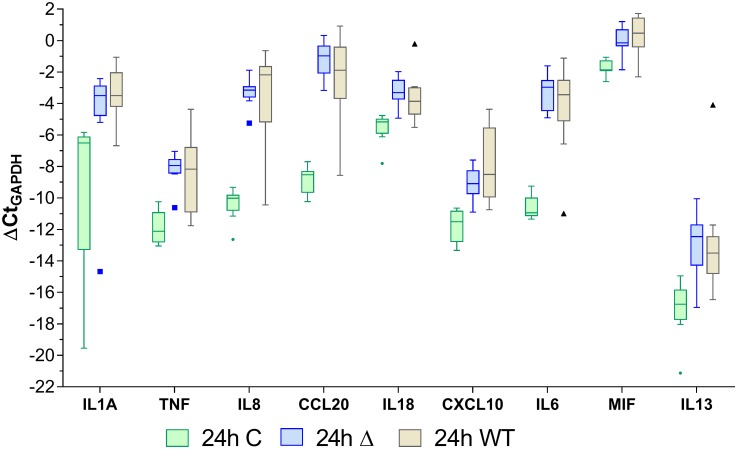
The relative mRNA expression of the stimulated cells and unstimulated controls at the 24h time point. Whiskers and outliers determined by the Tukey method. C = Control; Δ = PG343 biofilms; WT = W50 biofilms.

Array analysis of the RNA extracts from cells after stimulation showed that at 4h, relative to the unstimulated controls, the cells stimulated with the PG343 biofilms expressed a higher fold change of mRNA corresponding to the pro-inflammatory cytokines TNF-α, IL-1α, IL-6 and the chemokines IL-8 and CCL20 compared to the wild type, whereas the IL-18 and IL-13 mRNA levels did not show a marked change compared to controls and wild type (Fig 4). With respect to the unstimulated controls, the 24h stimulations yielded the highest relative fold changes in mRNA expression versus the 4h stimulations and there were differences in the cytokine expression profiles between the mutant and wild type stimulations at 24h (Fig 5). At 24h, cells stimulated by the mutant biofilm expressed a greater fold change of mRNAs for IL-18, IL-13 and CCL20 compared to the wild type biofilm, whereas IL-1α and CXCL10 were expressed to a greater level in the former. mRNA corresponding to other pro-inflammatory cytokines such as IL-8, IL-6 and TNF-α was found to be expressed at a similar level in both the mutant and wild type stimulations at 24h.

#### Interleukin-8, Interleukin-6 and tumor necrosis factor-α

Significantly higher amounts of the pro-inflammatory cytokines, IL-8 and IL-6 were measured in the supernatants of the W50 biofilm stimulations compared to the PG343 biofilms. The striking aspect of these data was that the supernatants of the PG343 biofilm stimulations contained reduced levels of IL-8 and IL-6 compared to their respective non-stimulated controls (Fig 3). However, the mRNA expression of these cytokines indicated a higher-fold upregulation of IL-8 and IL-6 transcripts in the cells stimulated with PG343 biofilms both at 4h and 24h (Figs 4 and 5). The ability of *P*. *gingivalis* to degrade IL-8, IL-6 and IL-1β has been documented in the literature and this may explain the decreased levels of these cytokines in the cell supernatants of the PG343 biofilm stimulations compared to W50, and the inverse relationship to their respective mRNA expression [36].

Differences were observed between the 4h and 24h time points amongst these cytokines. For example, detectable IL-8 levels were present at the 24h time point showing differences between the PG343 and W50 biofilms whereas, no IL-6 was detected at the 24h stimulations of both the biofilms (Fig 3). It is likely that the biofilms’ rate of IL-6 degradation is higher than IL-8 and this may relate to the fitness of the biofilm. While IL-8 is a chemokine that is primarily concerned with attracting neutrophils to an insulted site, IL-6 is thought to be a major regulator of the pro-inflammatory response towards an infectious agent, particularly in linking the innate and adaptive immune responses [37, 38]. By degrading IL-6, the oral biofilm may prevent IL-6 mediated signaling to recruit the Th2 and Th17 responses to more effectively address the microbial challenge.

Although little or no TNF-α was detected in the cell culture supernatants of both PG343 and W50 biofilms, mRNA expression for this potent cytokine suggested a more acute response by the cells toward the PG343 biofilms at 4h with both biofilm types showing equal stimulation of this cytokine transcription at 24h. *P*. *gingivalis* cysteine proteases are reported to degrade TNF-α rapidly (<10 mins), and the chosen time points for supernatant harvest (at 4h and 24h) is likely to be too late to observe the effect of the mRNA transcription as intact TNF-α in the cell culture supernatants [39].

#### Interleukin-1α and Interleukin-1β

The mRNA expression pattern and measured levels of IL-1α and IL-1β in supernatants suggests that biofilms may not degrade IL-1α unlike the pattern observed for IL-1β, where little or none were detected across all conditions at the 4h time point. At 24h however, elevated concentrations were observed when both biofilms were compared to unstimulated controls, but a reduced level was observed with regards to the PG343 biofilm compared to W50 suggesting the possibility of a higher degradation rate of IL-1β by the PG343 biofilm (Fig 3).

Studies have reported that *P*. *gingivalis* can antagonize production of IL-1α and IL1β when used to co-stimulate monocytes with other species in planktonic phase, while a non-degradative hypothesis is advanced with stimulation of *P*. *gingivalis* on its own with its LPS being identified as the main component of IL-1 stimulation [8, 40, 41]. Synergistic co-stimulation of oral keratinocytes is reported in the literature when the cells were stimulated with 3-species biofilms consisting of *S*. *gordonii*, *P*. *gingivalis* and *F*. *nucleatum* while secreted IL-1α levels are reported to be directly related to the amount of *F*. *nucleatum* cells present in the biofilms [42, 43]. The observed levels of IL-1α and IL-1β in the present study may be a result of stimulation by *F*. *nucleatum* and *P*. *gingivalis* and the mRNA data suggests that the PG343 containing biofilms may be exerting a greater pro-inflammatory effect compared to the W50 containing biofilms.

#### Interleukin-18, Interferon-Ɣ and ICAM-1

Transcription of IL-18 and IL-13 mRNA were downregulated by the PG343 biofilms at 4h compared to non-stimulated controls and W50 biofilms, but upregulated at 24h compared to control and W50 biofilms. IL-18 is thought to be a regulatory protein that promotes IFN-Ɣ production [44], and while it is notable that IFN-Ɣ levels in most of the samples measured were below detection limits at both 4h and 24h (S1 Fig), the regulatory IL-18 transcription suggests that more IFN-Ɣ production could be stimulated by the cells in response to the PG343 biofilms than the W50 biofilms. Indeed, IFN-Ɣ is known to promote production of ICAM1 and the concentration of ICAM1 detected in both the biofilm stimulation and controls were comparable, suggesting a possible block in IFN-Ɣ production by the biofilms, possibly by post-translational modulation as IFN-Ɣ suppression is reported of *T*. *denticola* and *P*. *gingivalis* [45–47].

#### Interleukin-13 and Latency Associated Peptide

A greater prevalence of Latency Associated Peptide (and by extension TGF-β) was observed in cells stimulated by the W50 biofilms compared to the PG343 and controls at both 4h and 24h, with the PG343 biofilm stimulations having similar levels as in controls (Fig 3). TGF-β and IL6 are believed to act in concert to differentiate naïve T-cells into an IL17 secreting Th17 lineage, and this data is consistent with the amount of IL6 detected in PG343 vs W50 biofilm stimulations, as increased presence of IL6 is thought to also induce TGF-beta production [48, 49]. IL-13 mRNA was found to be up regulated in the cells stimulated by the PG343 biofilms compared to W50 biofilms at 24h. *P*. *gingivalis* LPS is reported to stimulate a strong IL13 response in a mouse model [46] and though the cells used in the present study are not immune cells, stimulation of IL-13 mRNA transcription is still observed at both 4h and 24h with a greater effect shown by the PG343 biofilms. However, in terms of the actual cytokines detected, the opposite was observed between the PG343 and W50 biofilms at 4h (S1 Fig). It is possible that the biofilm degrades IL13, although no studies of this exist to the authors’ knowledge.

#### Macrophage migration inhibitory factor and CCL20

The expression of macrophage migration inhibitory factor (MIF) and CCL20 in the biofilm stimulations were elevated compared to controls but similar to each other. MIF was not measured by the immunoassay and it is also reported to be cleaved by Kgp; the mRNA expression in this study reveals a marginally higher stimulation at 4h by the PG343 biofilms compared to the W50 biofilms [50]. A similar pattern for CCL20 was also observed except the transcription of this chemokine was considerably elevated in relation to the unstimulated controls at both 4h and 24h. The CCL20 observations are consistent with data reported in the literature with regards to human primary gingival fibroblasts and *P*. *gingivalis* in planktonic phase [51].

## Conclusions

Taken together, these data suggest that the PG343 biofilms lacking in *P*. *gingivalis* methionine gamma lyase may have a more immunogenic phenotype as the cytokine levels and mRNA expression reveal a more acute pro-inflammatory effect compared to the biofilms containing the wild type W50 strain. However, the response elicited by the W50 biofilms could be more insidious, by way of the slower accumulation of the cytokines and a much more controlled inflammatory response at the 4h time point. The presence of elevated levels of intact cytokines such as IL-8, IL-6 and IL-1β in the biofilm-cell milieu may help activate inflammatory pathways that could promote microbial fitness in the oral cavity, possibly modelling the low grade inflammation that exists in the normal healthy gingiva. A role for the *P*. *gingivalis* enzyme methionine gamma lyase in influencing the inflammatory response of the oral mucosa by modelling the oral biofilm community composition as proposed by the keystone pathogen hypothesis is suggested.

## Supporting Information

S1 Fig Concentrations of E-selectin, IL-13, interferon-gamma and interferon-alpha detected in cell supernatants of the PG343 and W50 biofilm stimulations (n = 9 each). (TIF)
