# Supplementary figures and images for: In Vitro Effect of Porphyromonas gingivalis Methionine Gamma Lyase on Biofilm Composition and Oral Inflammatory Response
Source: PLoS One. 2016 Dec 29;11(12):e0169157. doi: 10.1371/journal.pone.0169157 (PMC5199072; doi:10.1371/journal.pone.0169157)

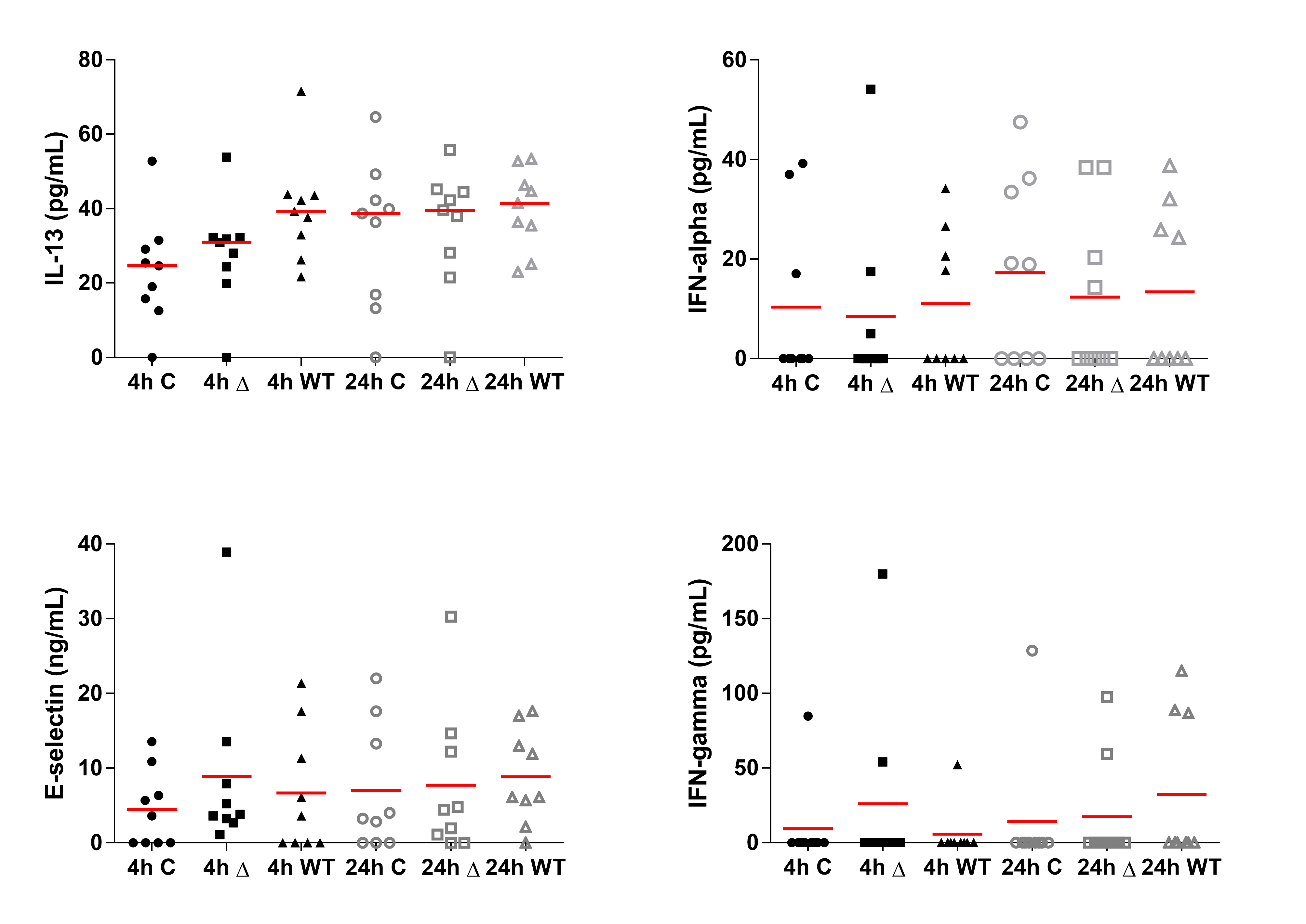

Supplement: S1 Fig — (TIF) [file pone.0169157.s001.tif]
